# High-Intensity Interval Training Improves Cognitive Flexibility in Older Adults

**DOI:** 10.3390/brainsci10110796

**Published:** 2020-10-29

**Authors:** Said Mekari, Heather F. Neyedli, Sarah Fraser, Myles W. O’Brien, Ricardo Martins, Kailey Evans, Meghan Earle, Rachelle Aucoin, Joy Chiekwe, Quinn Hollohan, Derek S. Kimmerly, Olivier Dupuy

**Affiliations:** 1 School of Kinesiology, Acadia University, 550. Main Street, Wolfville, NS B4P 2R6, Canada; rschultzmartins@gmail.com (R.M.); evansk3@myumanitoba.ca (K.E.); mearle@kinduct.com (M.E.); rachelle.aucoin@acadiau.ca (R.A.); joychiekwe@gmail.com (J.C.); quinn.mk.hollohan@gmail.com (Q.H.); 2 Division of Kinesiology, Faculty of Health, School of Health and Human Performance, Dalhousie University, Halifax, NS B3H 1T8, Canada; hneyedli@dal.ca (H.F.N.); myles.obrien@dal.ca (M.W.O.); dskimmerly@dal.ca (D.S.K.); 3 Interdisciplinary School of Health Sciences, University of Ottawa, Ottawa, ON K1N 74K, Canada; Sarah.Fraser@uottawa.ca; 4 Laboratory MOVE (EA 6314), Faculty of Sport Sciences, University of Poitiers, 86000 Poitiers, France; olivier.dupuy@univ-poitiers.fr

**Keywords:** aging, exercise intensity, cognition, brain health, cardiorespiratory fitness

## Abstract

**Introduction:** Regular aerobic exercise is associated with better executive function in older adults. It is unclear if high-intensity-interval-training (HIIT) elicits moderate-intensity continuous training (MICT) or resistance training (RT). We hypothesized that HIIT would augment executive function more than MICT and RT. **Methods:** Sixty-nine older adults (age: 68 ± 7 years) performed six weeks (three days/week) of HIIT (2 × 20 min bouts alternating between 15 s intervals at 100% of peak power output (PPO) and passive recovery (0% PPO); *n* = 24), MICT (34 min at 60% PPO; *n* = 19), or whole-body RT (eight exercise superior improvements in executive function of older adults than moderate-intensity-continuous-training, 2 × 10 repetitions; *n* = 26). Cardiorespiratory fitness (i.e., $\dot{V}O_{2max}$) and executive function were assessed before and after each intervention via a progressive maximal cycle ergometer protocol and the Stroop Task, respectively. **Results:** The $\dot{V}O_{2max}$ findings revealed a significant group by time interaction (*p* = 0.001) in which all groups improved following training, but HIIT and MICT improved more than RT. From pre- to post-training, no interaction in the naming condition of the Stroop Task was observed (*p* > 0.10). However, interaction from pre- to post-training by group was observed, and only the HIIT group exhibited a faster reaction time (from 1250 ± 50 to 1100 ± 50 ms; *p* < 0.001) in switching (cognitive flexibility). **Conclusion:** Despite similar improvements in cardiorespiratory fitness, HIIT, but not MICT nor RT, enhanced cognitive flexibility in older adults. Exercise programs should consider using HIIT protocols in an effort to combat cognitive decline in older adults.

## 1. Introduction

Advancing age is associated with a decline in higher order cognitive processes [1] known as executive functions (EFs), which are responsible for updating, shifting, and inhibiting cognitive actions [2]. These adverse age-related changes in cognitive functions increase older individuals’ risk of developing major neurocognitive disorders and diseases (e.g., dementia and Alzheimer’s disease) [3,4]. The Stroop Task is a commonly used assessment of executive function [5], and performance on this task predicts age-related cognitive decline [6]. The task consists of multiple conditions that vary in complexity, which permits the isolation of executive versus processing speed components (e.g., reading and responding) [7,8,9]. Importantly, executive functions are modifiable in older individuals [10], highlighting the importance of determining the impact of lifestyle interventions (e.g., exercise) on improving these higher-order cognitive processes in persons at risk for future neurocognitive disorders.

Engaging in regular physical activity and exercise has been associated with greater higher order cognitive functions in older adults. Specifically, cross-sectional and interventional studies have demonstrated that greater aerobic fitness and aerobic exercise favorably influence executive function in older adults [11,12]. However, the majority of exercise intervention studies have been conducted using moderate-intensity continuous training (MICT) protocols (as reviewed by Sáez de Asteasu et al. [13]). High-intensity interval training (HIIT) has been shown to improve cardiorespiratory fitness and vascular function to a greater extent than MICT in older adults [14,15], but whether HIIT enhances cognitive functions to a greater extent than MICT in this population is unclear since the number of studies is surprisingly limited [16,17]. However, several studies have suggested that high-intensity training could have a greater impact on our brain and that there would be a positive relationship between exercise intensity and brain adaptations to training.

Several exercise longitudinal studies have suggested that the intensity of aerobic training plays a particular role in improving the cognitive functions of the elderly. Van Gelder et al. [18] reported that older adults who exercised at a lower intensity (using questionnaire) were more likely to develop dementia 10 years later compared with those who exercised at a higher intensity. Angeraven et al. [19] also found that the average intensity of the weekly physical activities of middle aged and older adults are positively associated with cognitive performance. In addition, Brown et al. [20] indicated that the intensity, rather than the quantity, of physical activity might be more important to improve cognitive functions. From a neurophysiological point of view, a higher intensity of exercise provides a greater stimulus for the release of growth factors (e.g., brain-derived neurotrophic factor) [21], which are implicated in maintaining and improving several aspects of brain function [22,23]. Altogether, it is plausible that repeated high-intensity interval exercise translates to more favorable adaptations in higher-order cognitive functions than lower intensity exercise.

These previous findings provide some rationale to test the hypothesis that HIIT may provide an optimal strategy to enhance cognitive function in older adults. Recently, animal and human studies have provided findings with either null [24,25] or positive effects of HIIT on cognition [26,27,28,29,30,31]. Furthermore, Kovacevic et al. [27] found that 12 weeks of HIIT had a greater impact on cognitive function (e.g., working memory) than moderate-intensity continuous exercise in older adults. However, these results need to be confirmed because another study failed to observe an improvement in the incongruent condition (more executive condition) of the computerized Stroop Task following 16 weeks of HIIT (90:70% maximum heart rate at intervals of 4:3 min for 30 min) in cognitively healthy older adults [31]. Though these results are encouraging, there is still very little evidence that HIIT has superior effects on cognition when compared to moderate intensity exercise in older adults.

It has long been thought that only aerobic training and the improvement of cardiorespiratory capacity could explain the improvement in cognitive performance in the elderly, but other forms of exercise training may enhance cognitive function in older adults. Resistance training (RT) is a commonly recommended form of physical exercise for older adults, primarily as a means of preserving muscle and bone function [32]. As reviewed by Northey et al. [33], RT provides an effective stimulus to enhance the cognitive function of a magnitude similar to that of MICT among older adults. Most studies evaluating the impact of RT on cognition have been greater than 12 weeks in duration and/or in populations with mild cognitive impairments [13,33]. Though Berryman et al. [34] stipulated that “all roads lead to Rome,” there has been no study comparing HIIT, MICT, and RT on the several executive processes of older adults.

The primary purpose of the present study was to test the hypothesis that aerobic exercise training (HIIT or MICT) would improve cognitive function more than a non-aerobic RT group, and that HIIT would augment cognitive performances more than MICT and RT in older adults. The secondary purpose of the study was to test the hypothesis that cardiorespiratory fitness, as measured through $\dot{V}O_{2max}$ would be most improved following HIIT, and that HIIT and MICT would exhibit greater increases in aerobic fitness than the RT group.

## 2. Materials and Methods

### 2.1. Participants

Sixty-nine volunteer participants (46 females; 68 ± 7 years) were recruited from the Acadia Active Aging (AAA) Program. Prior to starting this study, participants were active three times a week through the AAA program. All participants were healthy and had normal-to-corrected vision. None of the participants had a history of neurological or psychiatric disorders, colour blindness, surgery with general anaesthesia during the previous 6 months, nor did they have involuntary tremors, epilepsy, or drug/alcohol problems. Participants were excluded if they (a) had any signs or symptoms of cardiovascular, pulmonary and/or metabolic diseases, (b) had a resting heart rate above 100 bpm or a resting blood pressure above 160/90 mmHg, (c) experienced orthopaedic or musculoskeletal problems that could affect their exercise ability, (d) score < 24 on the Mini-Mental State Examination (MMSE), and (e) if they were taking any medication that could affect cognitive function or heart rate. All criteria were assessed during a telephone screening and the first meeting at the research center with a certified exercise physiologist. The protocol was reviewed and approved by the Institutional Research Ethics Board in the Health Sciences of Acadia University (code: REB 15-09), and all participants provided written, informed consent.

### 2.2. Experimental Design

Participants were assessed in the laboratory before and after the exercise training interventions. At each visit, participants underwent a complete physical evaluation and a cognitive evaluation that included measurements of height, weight, MMSE, a computerized Stroop Task, and a maximal continuous graded cycle exercise test. In order to minimize known confounding influences during exercise testing, participants refrained from consuming caffeine or smoking within 2 h and drinking alcohol within 6 h of any testing, consistent with the exercise testing guidelines from the Canadian Society for Exercise Physiology (CSEP). Additionally consistent with the CSEP exercise testing guidelines, participants were asked to refrain from moderate (3–6 Metabolic Equivalent (METs)) to vigorous (>6 METs) exercise 24 h prior to their testing and were also asked to consume a normal breakfast on the morning of the test. No effort was made to control the nutrition of the participants. Participants were then randomly assigned to one of the three experimental groups: HIIT, MICT, or RT. We used a stratified randomization procedure [35] to ensure that the intervention groups were balanced at baseline for sex and fitness levels. All training was completed under the supervision of a trained exercise physiologist. All study visits were performed in a thermoneutral environment (21 °C).

### 2.3. Maximal Continuous Graded Exercise Test

The maximal continuous graded exercise test was performed on cycle ergometer (Lode B.V., Groningen, The Netherlands). The initial workload was set at 1 W/kg body mass. The workload was increased by 15 W every minute until voluntary exhaustion. Strong verbal encouragement was given throughout the test. The power of the last completed stage was considered as the peak power output (PPO, measured in W). The relative volume rate of oxygen uptake ($\dot{V}O_{2}$, in mL/min/kg) was determined continuously on a 30-s basis using an automated cardiopulmonary exercise system (Parvo Medics TrueOne 2400, Salt Lake City, UT, USA). Gas analyzers were calibrated before each test using a gas mixture of known concentrations (15% O_2_ and 5% CO_2_). The primary criterion for the attainment of $\dot{V}O_{2max}$ was a plateau in $\dot{V}O_{2}$ (change < 2.1 mL/min/kg) despite an increase in workload. In the absence of a plateau, the attainment of $\dot{V}O_{2peak}$ was based upon a respiratory exchange ratio of ≥1.10 and the inability to maintain a pedaling cadence of 60 revolutions/min. Furthermore, $\dot{V}O_{2max}$ was considered to be the highest $\dot{V}O_{2max}$ value attained during the test if the following criteria were observed: (1) a respiratory exchange ratio of ≥1.10 and (2) a peak heart rate ≥95% age-predicted maximum (i.e., 220 − age). Approximately 85% of the participants attained both criteria. Electrocardiographic activity was continuously monitored using a 12-lead electrocardiogram (ECG) (Philips, Netherlands). All tests were administered by a certified exercise physiologist.

### 2.4. Exercise Training Protocols

**Figure 1:**
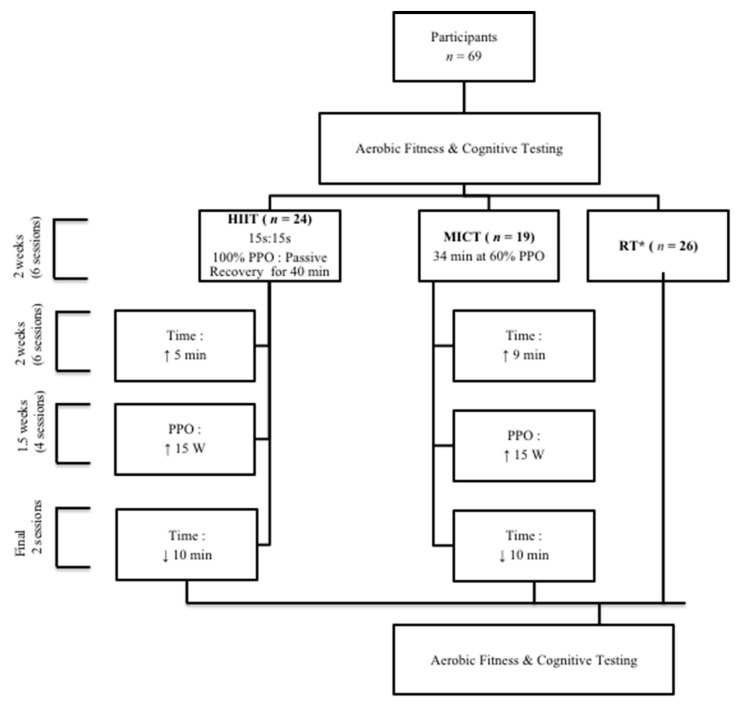
Schematic of the experimental design and each of the high-intensity interval training (HIIT), moderate-intensity continuous training (MICT), and whole-body resistance training (RT). PPO: peak power output (measured in watts). * The RT protocol consisted of 8 exercises targeting all major muscle groups for 2 sets of 10 repetitions.

For the three training protocols, all sessions were supervised and conducted 3 days per week (Mondays, Wednesdays, and Fridays) for 6 weeks. Eighty percent of the 18 sessions had to be completed to be eligible for post-testing (see Figure 1)

The HIIT session was based on previous studies that compared the time to exhaustion, participant preference, and time spent near the $\dot{V}O_{2max}$ of various interval protocols [17]. The group performed 15 s cycling intervals at 100% PPO with 15 s of passive recovery (stop cycling, 0% of PPO) between. The cycling intervals were performed for two sets of 20 min (40 min total), with 5 min of passive recovery between. This was performed for the first 2 weeks and then increased to a total of 45 min for the remaining 4 weeks. During the last 2 weeks, the intensity was increased by 15 W. The last 2 training sessions (tapering sessions) were performed at the same intensity for a total of 35 min. All exercise training sessions began with a 5 min warm-up and recovery at 25% PPO.

The MICT protocol was based on the American College of Sports Medicine (ACSM) physical activity guidelines that recommend at least 30 min of daily moderate aerobic physical activity (18). Continuous cycling at 60% PPO for 34 min was initially prescribed. This duration was adjusted to ensure that the MICT protocol was isoenergetic to the HIIT protocol based on the assumption that mechanical efficiency, aerobic fitness, and PPO were similar between groups; that 20 min at 100% PPO expends the same energy as 34 min at 60% PPO (MICT = 122 kJ vs. HIIT = 120 kJ for an individual with a PPO of 100 W). To support this assumption, the HIIT and MICT groups demonstrated similar pre-training $\dot{V}O_{2max}$ and PPO (both, *p =* 1.00). To accommodate the matched increase in energy expenditure, the total exercise time was prolonged to 39 min for the remaining 4 weeks, and power output was increased by 15 W for the final 2 weeks. For the final 2 MICT sessions, participants decreased their cycling time from 39 to 30 min in preparation for the post-training determination of maximal aerobic fitness. Both the HIIT and MICT protocols were identical to the protocols used by O’Brien et al. [36].

The RT training was also based on resistance training guidelines for older adults from the ACSM (8–10 exercises using major muscles with 8–12 repetitions of each exercise). Each RT session began and ended with 3 min of light cycling at 25% PPO. Thereafter, participants completed a total of 8 strength exercises, alternating between muscle groups. The exercises were primarily isokinetic machine-based and included the leg press, bench press, hamstrings curl, shoulder press, and leg extensions. Cable-exercises included seated row and latissimus pull-down, as well as bird-dogs (i.e., a core exercise that involves kneeling on the floor and simultaneously extending the hip while flexing the contralateral shoulder). Each participant performed 2 × 10 repetitions at 70% of perceived one repetition maximum (1 RM) for the first 2 weeks. Both sets of each exercise were performed before starting the next exercise with 30–60 s of rest between sets. Upper- and lower-body exercises were alternated. Participants were instructed to increase the number of repetitions to 12 ad libitum. Once participants were able to perform 2 × 12 repetitions, the resistance was proportionally increased to a weight that equated to 10 repetitions of their new estimated 70% of 1 RM with the assistance of the supervising Canadian Society of Exercise Physiology Certified Exercise Physiologist (CSEP-CEP).

### 2.5. Cognitive Testing

The executive functions and speed of processing abilities were assessed with a computerized modified Stroop Task [7,8,9]. This test included 3 conditions. In the first condition (naming), the participant had to read 1 of 4 possible words appearing on the screen: “RED,” “BLUE,” “YELLOW,” or “GREEN.” These words were displayed in the same colour as their meaning. The answers were mapped to the letters “u,” “i,” “o,” and “p” on a keyboard, which participants used to give their answers with the right hand. The mapping remained the same throughout the task. The order was “index finger—red,” “middle finger—green,” “ring finger—blue,” and “little finger—yellow.” This condition was the simplest condition and it provided a measure of speed of processing. The second block consisted of a classic inhibition task that required naming the font colour of a colour-word, the meaning of the word being incongruent with the colour of the font (e.g., the word “BLUE” displayed in green font). This condition was more difficult, as it required participants to inhibit their reading of the colour word and only respond to the colour of the font. In these first three blocks, a fixation cross appeared for 500 ms, followed by the word for 3000 ms. The third block consisted of a switching task, which was identical to the inhibition task, except a square appeared instead of the fixation cross that for 25% of the trials, and when the square appeared, participants were asked to read the colour-word instead of naming the font colour. The reading trials appeared randomly throughout the block, which made this condition one of cognitive flexibility as the participant had to remember the rules (square vs. fixation cross) and respond based on these rules. Each of the three blocks contained 60 trials, and the screen was blank between the trials. Before each condition, participants completed practice trials; 12 for the congruent condition, 12 for the inhibition condition, and 20 for the switching condition. During practice and experimental trials, a visual feedback (“error”) was given for incorrect responses only.

### 2.6. Data and Statistical Analyses

Relative $\dot{V}O_{2}$ data were averaged over 15 s intervals for the duration of the graded exercise protocol. Maximum or peak $\dot{V}O_{2}$ were considered as the greatest 30 s averaged $\dot{V}O_{2}$. Reaction times for each trial (60 trials per condition) were averaged for each of the Stroop Test conditions. All statistical analyses were done on SPSS v.25 for Mac. The significance level was set at *p* < 0.05 for all analyses. Standard statistical methods were used for the calculation of means and standard deviations. The normal Gaussian distribution of the data was verified by the Shapiro–Wilk test, and homoscedasticity was verified by a modified Levene’s Test. The compound symmetry, or sphericity, was checked by Mauchly’s test. When the assumption of sphericity was not met, the degree of freedom of F-ratios were adjusted according to the Greenhouse–Geisser procedure when the epsilon correction factor was <0.75, or according to the Huynh–Feldt procedure, when the epsilon correction factor was >0.75. For $\dot{V}O_{2max}$, a 3 group (HIIT, MICT, and RT) × 2 time (pre- vs. post-training) mixed ANOVA (α = 0.05) with group as the between-subjects factor and time as the within-subjects factor. For the Stroop Test, initially a 3 Stroop Task (naming, inhibition and switching) × 2 group (HIIT, MICT, and RT) × 2 time mixed ANOVA was conducted with group as the between subjects factor. To follow on a significant three-way interaction, separate 3 group × 2 time ANOVAS on each Stroop Task were run to determine if there was a significant two-way interaction that would indicate that the groups showed differential change in score pre- to post-training. Both the naming and inhibition task showed near ceiling effects in terms of accuracy (99.7% ± 0.6% correct and 98.7% ± 2.5% correct on average, respectively), so analysis was not run on these tasks. There was more variability in the accuracy scores for the switching task (89.7% ± 6.6%). An arcsine transformation was applied to the proportional data to account that the high end of the distribution was likely to be truncated by ceiling accuracy. The transformed proportion correct was analyzed by a 2 group (HIIT, MICT and RT) × 2 time mixed ANOVA. All post-hoc *t*-tests were Bonferroni-corrected for multiple comparisons3.

## 3. Results

### 3.1. Participant Characteristics

The descriptive characteristics of the participants can be found in Table 1.

### 3.2. Cardiorespiratory Fitness

**Figure 2:**
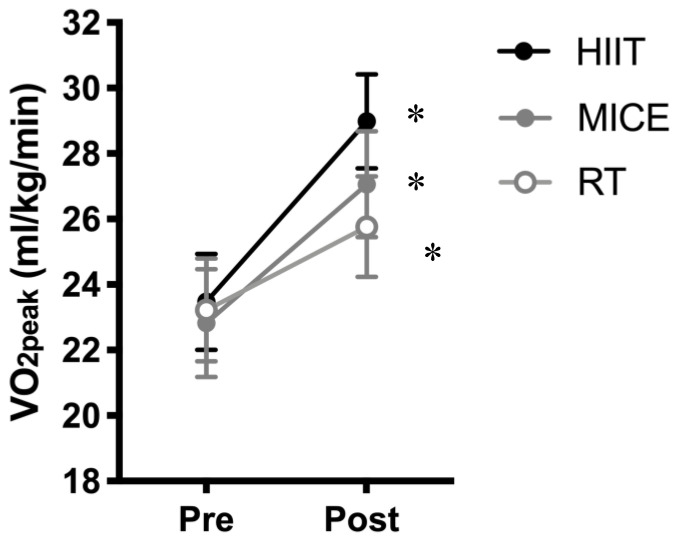
Comparison of changes in $\dot{V}O_{2max}$ from pre- to post-training following high-intensity interval training (HIIT), moderate-intensity continuous training (MICT), and whole-body resistance training (RT). Data are presented as means ± SEM. * different from pre-training (*p* < 0.05).

All data from the graded cycle exercise tests are presented in the Figure 2. First of all, we found no significant difference between groups on $\dot{V}O_{2max}$: *F*(3,68) = 0.53, *p* = 0.66, and *η^2^* = 0.02. There was a main effect of time—*F*(1,68) = 62.2, *p* < 0.001, and *η*^2^ = 0.48—but this was superseded by the group × time interaction, where *F*(3,68) = 6.05, *p* = 0.001, and *η*^2^ = 0.21. All intervention groups showed an increase in $\dot{V}O_{2max}$ (HIIT, *t*(23) = 9.10, *p* < 0.001, *d* = 1.86; MICT, *t*(18) = 4.90, *p* < 0.001, *d* = 1.12; RT, *t*(20) = 4.25, *p* < 0.001, and *d* = 0.92). However, examining the effect sizes showed that group × time interaction was driven by the HITT group having the greatest improvement in $\dot{V}O_{2max}$ from the pre to post-test, followed by the MICT, and then the RT groups.

### 3.3. Cognitive Task

**Figure 3:**
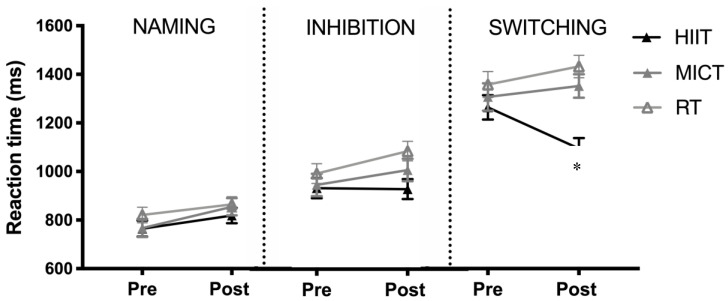
Comparison of changes in reaction time of the Stroop Task from pre- to post-training following high-intensity interval training (HIIT), moderate-intensity continuous training (MICT), and whole-body resistance training (RT). Data are presented as means ± SEM. * Group × time interaction, different from pre- training only for HIIT group (*p* < 0.05).

For the initial three-way ANOVA including group, Stroop Task, and timepoint, all main effect and two-way interactions were significant (*p* < 0.05) (Figure 3). Critically though, the three way interaction was significant, with *F*(3.12,101.3) = 5.67, *p* = 0.001, and *η*^2^ = 0.15. To follow up on the significant three-way interaction, separate group x time ANOVAs were run for each Stroop Task.

For the naming portion of the Stroop Task, there was no main effect of group on reaction time, with *F*(2,65) = 0.718, *p* = 0.49, and *η*^2^ = 0.02. The main effect of time was significant—*F*(1,66) = 18.43, *p* < 0.001, and *η*^2^ = 0.22—with response time increasing from the pre-training. The interaction between group and time was not significant, with *F*(2,65) = 0.794, *p* = 0.46, and *η*^2^ = 0.03, thus indicating that the increase in response time did not differ between groups.

For the interference portion of the Stroop Task, there was no main effect of group on reaction time, with *F*(2,65) = 2.07, *p* = 0.13, and *η*^2^ = 0.06. There was a main effect of time—with *F*(1,65) = 7.06, *p* = 0.01, and *η*^2^ = 0.10—in which the score increased from pre to post-training. Finally the two way interaction between group and timepoint was not significant, with *F*(2,65) = 2.47, *p* = 0.09, and *η*^2^ = 0.07.

For the switching portion of the Stroop Task, there was a main effect of group on reaction time (*F*(1,65) = 10.1, *p* < 0.001, and *η*^2^ = 0.24), but this was superseded by a higher order interaction. The main effect of timepoint was not significant, with *F*(1,65) = 0.704, *p* = 0.40, and *η*^2^ = 0.01. There was a significant interaction between group and timepoint, with *F*(2,65) = 11.0, *p* < 0.001, and *η*^2^ = 0.25 (Figure 3). There was a significant decrease in response time following training in the HIIT group only, with *t*(23) = 6.89, *p* < 0.001, and *d* = 1.06. No other group had a significant change in response time (MICT, *t*(18) = 0.90, *p* = 0.38, *d* = 0.19; RT, *t*(20) = 1.07, *d* = 0.20, and *p* = 0.15) (see Figure 3). Differences between groups were not due to a speed–accuracy trade-off, as there were no significant effects of group, time, or the interaction between them (all *p* > 0.30).

## 4. Discussion

The purpose of the present study was to test the hypothesis that aerobic exercise training (HIIT or MICT) would improve cognitive function more than a non-aerobic RT group and that HIIT would augment cognitive performances more than MICT and RT in older adults. Consistent with our hypothesis, HIIT favorably improved reaction time in the switching condition of the Stroop Task. However, neither MICT nor RT improved Stroop Task performance in this group of cognitively healthy older adults. In line with our hypothesis, cardiorespiratory fitness was more enhanced following HIIT than it was following MICT and RT. Our findings demonstrate that short-term HIIT has the capacity to result in meaningful improvements in executive function, whereas other frequently implemented training protocols failed to elicit such beneficial adaptations in an older population.

A considerable amount of previous research has sought to determine if exercise-training interventions modify cognitive function in older populations [12,13,33]. Despite this, limited studies have evaluated the impact of engaging in higher-intensity interval training on cognition. Adding to the current literature, our HIIT, but not MICT or RT protocols, elicited a sufficient stimulus for augmenting executive function in older adults (see Figure 3). Specifically, HIIT resulted in an enhanced, faster reaction time during the switching condition of the Stroop Task. Executive function declines with ageing, and impairments in these processes may be an early sign of the development and progression of cognitive diseases. We demonstrated that as little as six weeks of HIIT elicited improvements in higher-order cognitive functions that may combat the age-associated decline in these functions. Given that it is well-established that engaging in HIIT is safe for healthy older adults and that this population appears to enjoy this type of exercise training more than MICT [37,38], our findings suggest that exercise training programs aimed at enhancing or preventing the decline in cognitive function should incorporate HIIT-based protocols among older individuals.

Our findings support previous work conducted with children [28], adolescents [26], young adults [30,39], healthy older adults [27], and those who have had a stroke [29]. Additionally, our results on switching cognitive performance are consistent with those reported by Paliesen et al. [29] and Mekari et al. [39]. Indeed, these authors reported a positive effect of HIIT only on Trail B and not on Trail A. These results confirm the idea that more executive functions are sensitive to physical training and to aerobic improvement. Additionally, our cognitive flexibility data are consistent with similar processes reported by Jeaon et al. [26] in adolescents. However, none of these studies compared high-intensity training with moderate-intensity training and often used an active control group for comparison. Only Mekary et al. [39] found similar results on the switching condition of the same Stroop Task in young adults comparing interval training to continuous training. Specifically, young adults improved cognitive performance in the same switching task following HIIT (ES = 0.7). Furthermore, our study is the only one to confirm the superior effect of HIIT training on flexibility cognitive performance in older adults. This enhanced effect was recently reported in an elderly subject on memory functions. Kovacevic et al. [39] reported that 12 weeks of HIIT training improved selective memory function in healthy older adults. Interestingly, Costoe et al. [39] reported no positive effect of 12 weeks of HIIT on the inhibitory process (using the Stroop Task) in healthy older adults. Our results are partially in accordance with these results because we found no changes in reaction time during the inhibition task. Furthermore, we observed a greater effect of HIIT on cognitive flexibility than inhibition. This suggests that aerobic fitness may have a greater impact on the most challenging and complex cognitive processes. The lack of positive results reported by Cotsoe et al. was probably due to several reasons. This study was limited by a small sample size (HIIT: *n* = 11), and the complex (i.e., incongruent) condition implemented in their study consisted of 24 trials with only two response options (using right and left arrow keys), which may not have been challenging enough to test executive function, as exhibited by the short, similar reaction times (neural condition: ~25–30 s; incongruent: 35–40 s). It is likely that their relatively simple incongruent condition may have been largely dependent upon non-executive aspects of cognitive function (e.g., processing speed).

Our laboratory and others have previously demonstrated that greater cardiorespiratory fitness is positively associated with enhanced cognitive function in older adults [9,40]. Specifically, cognitive flexibility seems to be very sensitive to cardiorespiratory changes. In a cross-sectional study, Dupuy et al. [9] reported that more fit women (e.g., greater $\dot{V}O_{2max}$) displayed better reaction times in the same cognitive flexibility paradigm using the Stroop Task. A longitudinal study from Pedrovan et al. [41] found the same results after aerobic training.

Our HIIT and MICT groups exhibited greater enhancements in $\dot{V}O_{2max}$ following training than the RT group. Given that the aerobic and resistance training protocols produced improvements in $\dot{V}O_{2max}$ but only HIIT improved executive function, it appears that other factors beyond changes in cardiorespiratory fitness are responsible for enhancements in executive function in older populations. Though we were able to rule out cardiorespiratory fitness as a contributing factor to the enhancements in executive function, we were unable to determine the precise mechanisms responsible for our observations. In comparison to MICT, HIIT has been shown to provide a greater stimulus for the synthesis of brain-derived neurotrophic factor (BDNF), which is neurotrophic factor largely implicated in cognitive processes [21]. Furthermore, individuals with cognitive decline typically have lower levels of insulin-like growth factor 1 (IGF-1) [42]. Previous research by Herbert et al. [43] demonstrated that six weeks (one session every five days) of HIIT (6 × 30 s at ~140% of PPO interspersed with three min of active recovery) increased systemic IGF-1 levels in physically inactive older men. It is plausible that our HIIT protocol increased BDNF and/or IGF-1 to a greater extent than MICT or RT. Additionally, little is known about the impact of HIIT on the cerebrovasculature and how this relates to changes in cognitive function (see the work of Lucas et al. [17] for review). Recently, Guadagni et al. [44] demonstrated that six months of MICT (three days per week of 20–40 min at 30–70% heart rate reserve) augmented executive function in older adults and that this improvement was associated with improved cerebrovascular regulation. In the present study, larger blood flow responses and fluctuations in metabolic intensity in response to HIIT may have resulted in greater changes in cerebrovascular function than MICT and RT in six weeks, and these beneficial effects could translate to favourable improvements in higher-order cognitive functions. Future mechanistic studies are needed to determine the precise mechanisms responsible for the HIIT-induced improvements in executive function.

Though this study had a number of strengths including multiple exercise training protocols and direct measures of cardiorespiratory fitness in a large sample (*n* = 69) of older adults, it was not without limitations. Though widely used in the literature, we recognize that the MMSE has some limitations and does not replace a more rigorous neuropsychological testing battery when it comes to screening for dementia. We also acknowledge that our study focused only on the executive components of the computerized Stroop Task. Future studies that are able to administer a comprehensive cognitive battery could generate more information and help us in understanding the role of HIIT on all cognitive function in older adults. Furthermore, our study did not include additional measures that would provide mechanistic insight into our observed findings. Importantly, the study was not designed to uncover such information; it was instead designed to add to the current literature in that it demonstrated that HIIT provides greater enhancements in cognitive function than other frequently implemented training protocols (here being MICT and RT) in older adults. Additionally, exercise training studies in which samples are primarily comprised of older females demonstrated greater improvements in executive function than studies comprised of older males (as reviewed in Barha et al. [45]). Though our study groups consisted of a combination of older males and females, the proportion of each sex was similar in each group (%♀: HIIT = 63%; MICT = 53%; RT = 73%). More importantly, statistically controlling for sex did not influence the outcomes of the present study. Additionally, in this study, the lack of positive results of RT on cognitive performance could have been due to the relatively short duration protocol, which was not sufficient to induce neurophysiological and cognitive changes.

## 5. Conclusions

The findings of this study demonstrate that six weeks of HIIT were superior to MICT and RT at eliciting improvements in executive function in cognitively healthy older adults. HIIT produced enhanced improvements in executive function in the short-term, which may help to combat the negative influence of age on cognitive function and reduce the risk of future cognitive impairments. Exercise training programs aimed at enhancing executive function in older adults should consider implementing a higher-intensity and shorter duration form of HIIT, which may be the most effective program at improving executive function in this population.

## Figures and Tables

**Table 1:** Characteristics of participants.

	HIIT			MICT			RT		
	PRE	POST	ES	PRE	POST	ES	PRE	POST	ES
Sex	15 F, 9 M		--	10 F, 9 M		--	17 F, 9 M		--
Age	67 ± 5		--	68 ± 7		--	67 ± 7		--
Height (m)	1.6 ± 0.1		--	1.7 ± 0.1		--	1.6 ± 0.1		--
Weight (kg)	73 ± 12	73 ± 11	0.0	75 ± 13	75 ± 13	0.0	74 ± 12	75 ± 14	0.0
BMI (kg/m^2^)	26 ± 4	26 ± 4	0.0	26 ± 4	26 ± 4	0.0	26 ± 7	27 ± 4	0.0
MMSE	29 ± 1			29 ± 1			28 + 1		

Note: m: meters; kg: kilograms; BMI: body mass index; F: female; M: male; ES: effect size; MMSE: Mini-Mental State Examination.
